# Postnatal Antioxidant and Anti-inflammatory Treatments Prevent Early Ketamine-Induced Cortical Dysfunctions in Adult Mice

**DOI:** 10.3389/fnins.2020.590088

**Published:** 2020-11-04

**Authors:** Maria Bove, Paolo Tucci, Stefania Dimonte, Luigia Trabace, Stefania Schiavone, Maria Grazia Morgese

**Affiliations:** Department of Clinical and Experimental Medicine, University of Foggia, Foggia, Italy

**Keywords:** celastrol, indomethacin, ketamine, prefrontal cortex, redox, inflammation, animal models

## Abstract

Early brain insult, interfering with its maturation, may result in psychotic-like disturbances in adult life. Redox dysfunctions and neuroinflammation contribute to long-term psychiatric consequences due to neurodevelopmental abnormalities. Here, we investigated the effects of early pharmacological modulation of the redox and inflammatory states, through celastrol, and indomethacin administration, on reactive oxygen species (ROS) amount, levels of malondialdehyde (MDA) and antioxidant enzymes (superoxide dismutase 1, SOD1, glutathione, GSH, and catalase, CAT), as well as of pro-inflammatory cytokines (tumor necrosis factor-alpha, TNF-α, interleukin-6, IL-6, and interleukin-1 beta, IL-1β), in the prefrontal cortex of adult mice exposed to a neurotoxic insult, i.e. ketamine administration, in postnatal life. Early celastrol or indomethacin prevented ketamine-induced elevations in cortical ROS production. MDA levels in ketamine-treated mice, also administered with celastrol, were comparable with the control ones. Indomethacin also prevented the increase in lipid peroxidation following early ketamine administration. Whereas no significant differences were detected in SOD1, GSH, and CAT levels between ketamine and saline-administered mice, celastrol elevated the cortical amount of these antioxidant enzymes and the same effect was induced by indomethacin *per se*. Both celastrol and indomethacin prevented ketamine-induced enhancement in TNF-α and IL-1β levels, however, they had no effects on increased IL-6 amount resulting from ketamine exposure in postnatal life. In conclusion, our data suggest that an early increase in cortical ROS scavenging and reduction of lipid peroxidation, via the enhancement of antioxidant defense, together with inhibition of neuroinflammation, may represent a therapeutic opportunity against psychotic-like disturbances resulting, later in life, from the effects of a neurotoxic insult on the developing brain.

## Introduction

Early insults affecting the central nervous system (CNS) during crucial phases of its maturation have been reported to induce neurodevelopmental abnormalities. This has been associated with increased risk of developing psychotic-like disturbances in adult life (Hussain and Murray, 2015). In this pathological process, the prefrontal cortex (PFC), characterized by highly vulnerable cellular populations, has been described as one of the most consistently implicated brain regions (Selemon and Zecevic, 2015).

Multiple molecular mechanisms underlying long-term psychiatric consequences of early brain insults have been proposed. Among them, dysfunctions of the antioxidant enzymes, such as superoxide dismutase 1 (SOD1), glutathione (GSH), and catalase (CAT), have been described (Cabungcal et al., 2013). The expression and activity of these enzymes physiologically occur during key neurodevelopmental phases. Indeed, SOD1, expressed primarily in cortical neurons (Peluffo et al., 2005), has been shown to reach a peak in the second postnatal week (Ceballos-Picot et al., 1992). Similarly, CAT activity in rodent developing CNS has been found to be higher than in the mature brain (Del Maestro and McDonald, 1987; Hamby-Mason et al., 1997), with a maximum observed from postnatal day (PND) 5 to PND 10 (Del Maestro and McDonald, 1987; Aspberg and Tottmar, 1992). Moreover, GSH has been reported to increase and modify the redox state of the cells toward a more reduced condition starting from PND 10 until PND 30 (Galkina et al., 2017). Altered antioxidant defense in the brain may result in increased levels of reactive oxygen species (ROS) and consequent lipid peroxidation in neurons. One of the final products of this biochemical process is malondialdehyde (MDA). Enhanced amount of this highly reactive compound has been reported in the PFC of young mice perinatally exposed to a neurotoxic insult (Del Rio et al., 2005; Tsikas, 2017). The natural compound celastrol, derived from the root of *Tripterygium wilfordii*, pharmacologically modulate ROS amount and antioxidant defense system. It has shown to be effective for a broad range of pathological conditions, including neurodegenerative disorders (Kiaei et al., 2005; Paris et al., 2010; Choi et al., 2014), cerebral ischemia (Li et al., 2012; Jiang et al., 2018) and traumatic brain injury (Eroglu et al., 2014). Moreover, it has been reported to prevent psychotic-like behavioral alterations, oxidative stress and inflammatory imbalance in adult mice exposed to a neurotoxic insult in their postnatal life (Schiavone et al., 2019).

Neuroinflammation is a crucial contributor of long-term psychiatric consequences of early neurodetrimental insults. In particular, the developing brain is characterized by increased vulnerability to proinflammatory cytokines, such as Tumor necrosis factor (TNF)-α, interleukin-6 (IL-6), and interleukin-1 beta (IL-1β) (Hagberg and Mallard, 2005). In this regard, levels of TNF-ɑ were enhanced in the cerebellum of adult mice postnatally exposed to a neurotoxic insult (Schiavone et al., 2019). In addition, non-steroidal anti-inflammatory drugs (NSAIDs) have been shown to exert protective effects on neurodevelopmental processes. This occurs via the inhibition of the synthesis of inflammatory mediators at systemic level and via cyclooxygenase (COX) inhibition at blood brain barrier site (Favrais et al., 2007). Among NSAIDs, indomethacin, a non-selective inhibitor of COX 1 and 2, has been shown to readily pass the blood brain barrier (Parepally et al., 2006; Novakova et al., 2014). It also exerted neuroprotective effects in newborn rodents exposed to hypoxic-ischemic insult (Tutak et al., 2005; Taskin et al., 2009). Accordingly, clinical evidence showed that indomethacin, unlike ibuprofen, might be neuroprotective against the long term effects of cerebral insults, such as ventricular hemorrhage (Favrais et al., 2014).

Postnatal administration of subanesthetic doses of ketamine, a NMDA receptor (NMDA-R) antagonist, is a reliable tool to mimic in rodents an early insult interfering with brain maturation (Frohlich and Van Horn, 2014). In this regard, NMDA-Rs reach their maximum expression in the first 2 weeks of postnatal life. Hence, inhibition of these receptors in this period is associated with increased neuronal damage (Bubenikova-Valesova et al., 2008). Indeed, ketamine exposure during CNS development has been shown to cause a down-regulation of NMDA-Rs located in the PFC resulting in psychiatric-like symptoms in rat adult offspring (Ren et al., 2019). Furthermore, early genetic ablation or ketamine-induced blockade of NMDA-Rs of cortical parvalbumin-expressing GABAergic interneurons can induce in adult animals persistent behavioral deficits, reminiscent of cognitive and negative psychotic symptoms (Jeevakumar et al., 2015). In addition, we have demonstrated that ketamine administration at PNDs 7, 9 and 11 caused psychotic-like neurochemical and behavioral alterations in adult mice (Schiavone et al., 2019, 2020).

Here, we assessed the effects of early pharmacological modulation of the redox and inflammatory states, through celastrol and indomethacin administration, on possible alteration of ROS production, lipid peroxidation, as well as SOD1, GSH, and CAT levels in the PFC, induced by the exposure to an early neurotoxic trigger, i.e., ketamine, in postnatal life. Moreover, celastrol or indomethacin effects on possible ketamine-induced changes of proinflammatory cytokines levels, i.e., TNF-α, IL-6, and IL-1β were also assessed in the same brain region.

## Materials and Methods

### Animals

Mice were housed at constant room temperature (22 ± 1°C) and relative humidity (55 ± 5%), under a 12 h light/dark cycle (lights on from 7:00 AM to 7:00 PM). They had free access to food and water. Experimental procedures involving animals and their care were performed in conformity with the institutional guidelines of the Italian Ministry of Health (D.Lgs. n. 26/2014), the Guide for the Care and Use of Laboratory Animals: Eight Edition, the Guide for the Care and Use of Mammals in Neuroscience and Behavioral Research (National Research Council, 2004), the Directive 2010/63/EU of the European Parliament and of the Council of 22 September 2010 on the protection of animals used for scientific purposes, as well as the ARRIVE guidelines. The experimental protocol was approved by the Italian Ministry of Health (approval number 679/2017-PR, protocol n. B2EF8.17) Animal welfare was daily monitored throughout the experimental phase. All efforts were made to minimize the number of animals used, as well as their suffering.

### Experimental Protocol

A total of five C57/Bl6 male mice of 8–10 weeks of age, weighting 25–30 g, and ten age and weight-matched adult females (Envigo, San Pietro al Natisone, Italy) were mated (one male and two females per cage). Male pups were divided into the following experimental groups, according to the different treatments they received at PNDs 7, 9, and 11:

(1) Saline (10 ml/kg i.p.);
(2) Ketamine (Sigma-Aldrich Corporation, Saint Louis, MO, United States; 30 mg/kg i.p., dissolved in saline) (Sorce et al., 2010; Jeevakumar et al., 2015);
(3) Celastrol (Sigma Aldrich, Milano, Italy; 1 mg/kg i.p., dissolved in 50% DMSO/PBS) (Paris et al., 2010; Schiavone et al., 2019);
(4) A 50% DMSO/PBS solution (5 ml/kg i.p.);
(5) Ketamine (30 mg/kg i.p., dissolved in saline, injected in the right side of the peritoneum) and celastrol (1 mg/kg i.p., dissolved in 50% DMSO/PBS, injected in the left side of the peritoneum) (Schiavone et al., 2019)-indicated throughout the text as “ketamine + celastrol”;
(6) Indomethacin [Promedica, Parma, Italy, 10 mg/kg i.p., (La Vitola et al., 2018), dissolved in saline];
(7) Ketamine (30 mg/kg i.p., dissolved in saline, injected in the right side of the peritoneum) and indomethacin (10 mg/kg i.p., dissolved in saline, injected in the left side of the peritoneum) – indicated throughout the text as “ketamine + indomethacin”.

For ethical reasons, in keeping with the pursuing of 3R requirements foreseen by the Directive 2010/63/EU of the European Parliament, as well as of the Council of 22 September 2010 on the protection of animals used for scientific purposes, the ARRIVE guidelines, and also based on our previous experience (we did not detect any differences between a double with respect to single injection of vehicles), the group consisting of double-vehicle injection was omitted from the experimental protocol.

All pups were grown until adulthood (10 weeks of age). At this time point, they were euthanized by cervical dislocation for PFC collection.

### PFC Collection

The PFC of 10-weeks mice was collected by using the Mouse Brain Matrix, making coronal sections of 1 mm of thickness and dissecting it from the obtained brain slices according to the Mouse Brain in Stereotaxic Coordinates, 3rd Edition, Franklin and Paxinos (2015). Immediately after, tissues were frozen in isopentane and stored at −80°C, until biomolecular analyses were performed (Bove et al., 2018).

### ROS Measurement

Reactive oxygen species measurement in PFC was performed as previously described (Baek et al., 2018; Pirozzi et al., 2020), by using the fluorogenic dye 2′,7′dichlorofluorescein diacetate (Sigma Aldrich, Milano, Italy) (Kirkland et al., 2007). Briefly, tissue was homogenized in PBS 1× (pH = 7.4) according to the following proportion: 500 μl of PBS 1× for 2,5 mg of tissue. The dye was added to the sample with a final concentration of 5 μM and incubation was performed for 15 min at 37°C. Samples were than centrifuged for 10 min at 4°C and 12,500 rpm. The pellet was resuspended in 5 ml PBS 1× and put in ice for 10 min. After a 1-h incubation at 37°C, samples were analyzed in 96-well microplate by using a fluorometer (Filter Max F5, Multi-Mode Microplate Reader, excitation length 475 nm, emission length 535 nm). Results were expressed as μmol DCF/mg of tissue.

### MDA Assay

MDA assay was performed by using a commercially available kit (Sigma-Aldrich, Milano, Italy) as previously described (Fan et al., 2019), according to the manufacturer’s instructions. Each sample and standard analysis was performed in duplicate to avoid intra-assay variations.

### Enzyme-Linked Immunosorbent Assays

Samples were homogenized in 10 volumes of PBS with protease inhibitors, as previously described (Schiavone et al., 2019). Commercially available Enzyme-Linked Immunosorbent Assay (ELISA) kits were used for measurement of SOD1 (Wuhan Fine Biotech Co., Ltd.-FineTest, Wuhan, China), GSH (Biomatik Life Science Products and Service, Ontario, Canada), CAT (Wuhan Fine Biotech Co., Ltd.-FineTest, Wuhan, China), TNF-ɑ (MyBiosource, San Diego, CA, United States), IL-6 (MyBiosource, San Diego, CA, United States) and IL-1ß (MyBiosource, San Diego, CA, United States) in the PFC, according to the manufacturer’s instructions. All samples and standards were analyzed in duplicate to avoid intra-assay variations.

### Blindness of the Study

Data analysis was performed by researchers who were blind with respect to the treatment conditions. The blindness of the study was maintained until data analysis ended.

### Statistical Analysis

Statistical analysis was performed by using GraphPad 5.0 software for Windows. Data were checked for normality by using Bartlett’s test and then analyzed by One Way ANOVA, followed by Tukey’s *post hoc* test or Kruskal-Wallis test, followed by Dunn’s multiple comparison test. For all tests, a *p*-value < 0.05 was considered as statistically significant. Results are expressed as means ± mean standard error (SEM). No significant differences in all the considered parameters were detected between saline and 50% DMSO/PBS-treated animals. Therefore, graphs only include results related to saline-treated animals.

## Results

### Effects of Early Celastrol or Indomethacin Administration on ROS Production and Lipid Peroxidation in the PFC of Adult Mice Treated With Ketamine in Postnatal Life

**FIGURE 1:**
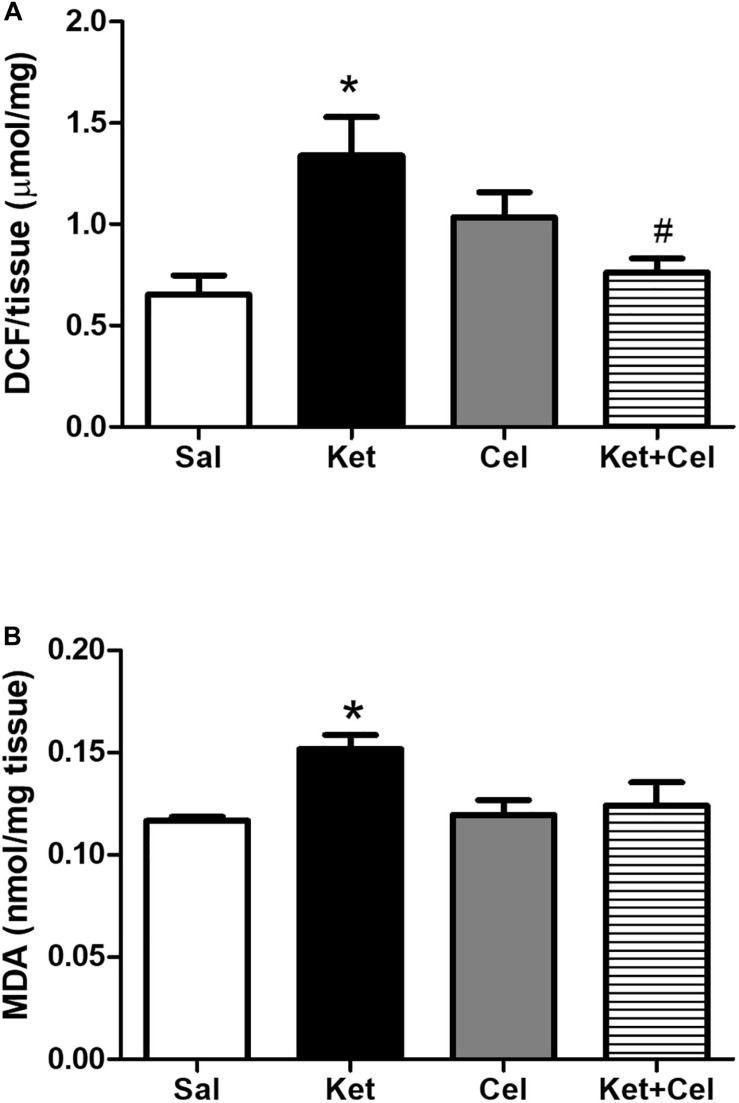
Effects of early celastrol administration on ROS production and MDA levels in the PFC of adult mice, administered with ketamine in postnatal life. **(A)** ROS production (μmol DCF/mg of tissue) in the PFC of 10 weeks mice receiving saline (Sal, *n* = 4) or ketamine (Ket, *n* = 4) or celastrol (Cel, *n* = 4) or ketamine + celastrol (Ket + Cel, *n* = 4) at PNDs 7, 9, and 11. One way ANOVA, followed by Tukey’s Multiple Comparison *post hoc* test, *F*_(3,12)_ = 5.742, *p < 0.05 Ket vs Sal, ^#^*p* < 0.05 Ket + Cel vs Ket. **(B)** MDA levels (nmol/mg tissue) in the PFC of 10 weeks mice receiving saline (Sal, *n* = 5) or ketamine (Ket, *n* = 8) or celastrol (Cel, *n* = 5) or ketamine + celastrol (Ket + Cel, *n* = 6) at PNDs 7, 9, and 11. Kruskal-Wallis test, followed by Dunn’s multiple comparison test, Kruskal-Wallis statistic = 9.573 *p < 0.05 Ket vs Sal.

**FIGURE 2:**
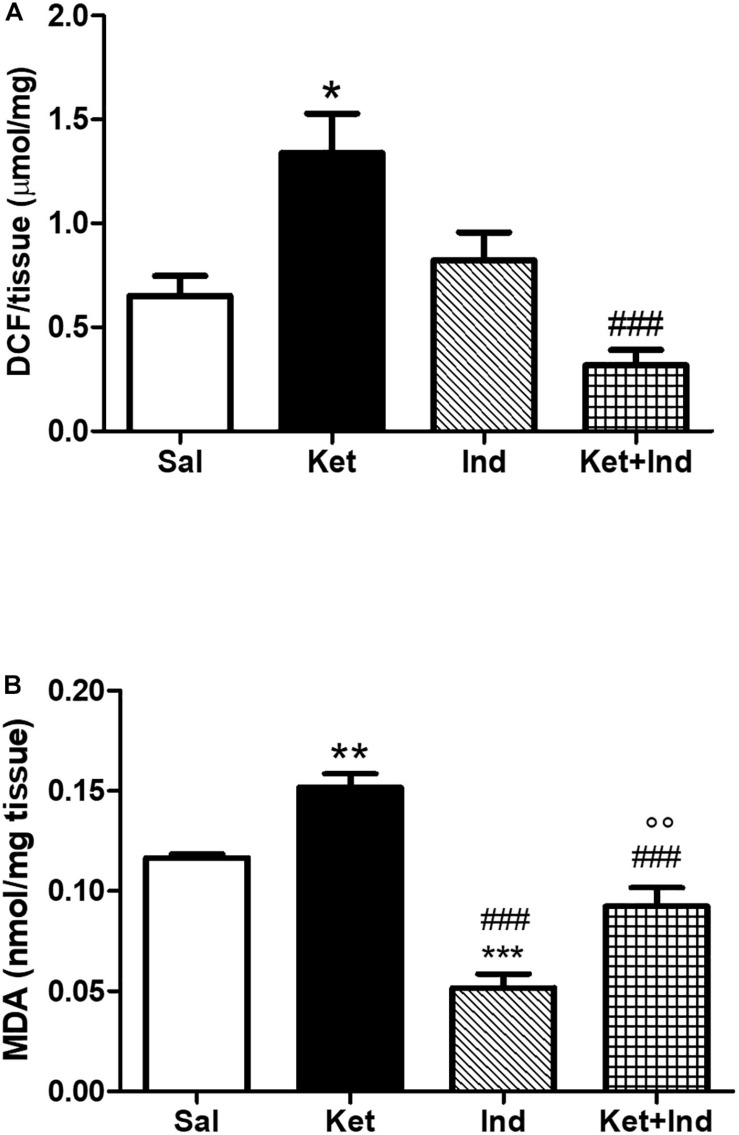
Effects of early indomethacin administration on ROS production and MDA levels in the PFC of adult mice, administered with ketamine in postnatal life. **(A)** ROS production (μmol DCF/mg of tissue) in the PFC of 10 weeks mice receiving saline (Sal, *n* = 4) or ketamine (Ket, *n* = 4) or indomethacin (Ind, *n* = 5) or ketamine + indomethacin (Ket + Ind, *n* = 4) at PNDs 7, 9, and 11. One way ANOVA, followed by Tukey’s Multiple Comparison *post hoc* test, *F*_(3,13)_ = 9.642, *p < 0.05 Ket vs Sal, ^###^*p* < 0.001 Ket + Ind vs Ket. **(B)** MDA levels (nmol/mg tissue) in the PFC of 10 weeks mice receiving saline (Sal, *n* = 5) or ketamine (Ket, *n* = 8) or indomethacin (Ind, *n* = 4) or ketamine + indomethacin (Ket + Ind, *n* = 5) at PNDs 7, 9, and 11. One way ANOVA, followed by Tukey’s Multiple Comparison *post hoc* test, *F*_(3,18)_ = 35.10, **p < 0.01 Ket vs Sal, ***p < 0.001 Ind vs Sal, ^###^*p* < 0.001 Ind vs Ket and Ket + Ind vs Ket, °°*p* < 0.01 Ket + Ind vs Ind.

To assess possible effects of early celastrol or indomethacin administration on ROS production and lipid peroxidation induced by ketamine exposure in postnatal life, we quantified ROS, and MDA levels in the PFC of adult mice. No significant differences in cortical ROS production were detected between controls and celastrol-injected animals. Ketamine exposure in postnatal life resulted in increased ROS production compared to saline-treated mice which was prevented by ketamine + celastrol administration (Figure 1A, One way ANOVA, followed by Tukey’s Multiple Comparison *post hoc* test, *F*_(3,12)_ = 5.742, *p* < 0.05). While no significant differences in MDA levels were observed between animals administered with saline and the celastrol group, adult mice that received ketamine in early postnatal life, showed significant MDA elevations in the considered brain region. Early celastrol administration was able to prevent ketamine-induced lipid peroxidation (Figure 1B, Kruskal-Wallis test, followed by Dunn’s multiple comparison test, Kruskal-Wallis statistic = 9.573, *p* < 0.05). Early ketamine + indomethacin administration prevented ketamine-induced elevation in ROS levels (Figure 2A, One way ANOVA, followed by Tukey’s Multiple Comparison *post hoc* test, *F*_(3,13)_ = 9.642, *p* < 0.05, and *p* < 0.001). Indomethacin treatment *per se* was able to significantly lower MDA levels with respect to both controls and ketamine-exposed mice. Lipid peroxidation in mice early receiving ketamine + indomethacin was decreased compared to ketamine-treated mice but did not reach the same levels than the ones detected in mice early exposed to indomethacin alone (Figure 2B, One way ANOVA, followed by Tukey’s Multiple Comparison *post hoc* test, *F*_(3,18)_ = 35.10, *p* < 0.01 and *p* < 0.001).

### Effects of Early Celastrol or Indomethacin Administration on Antioxidant Enzyme Expression in the PFC of Adult Mice Treated With Ketamine in Postnatal Life

**FIGURE 3:**
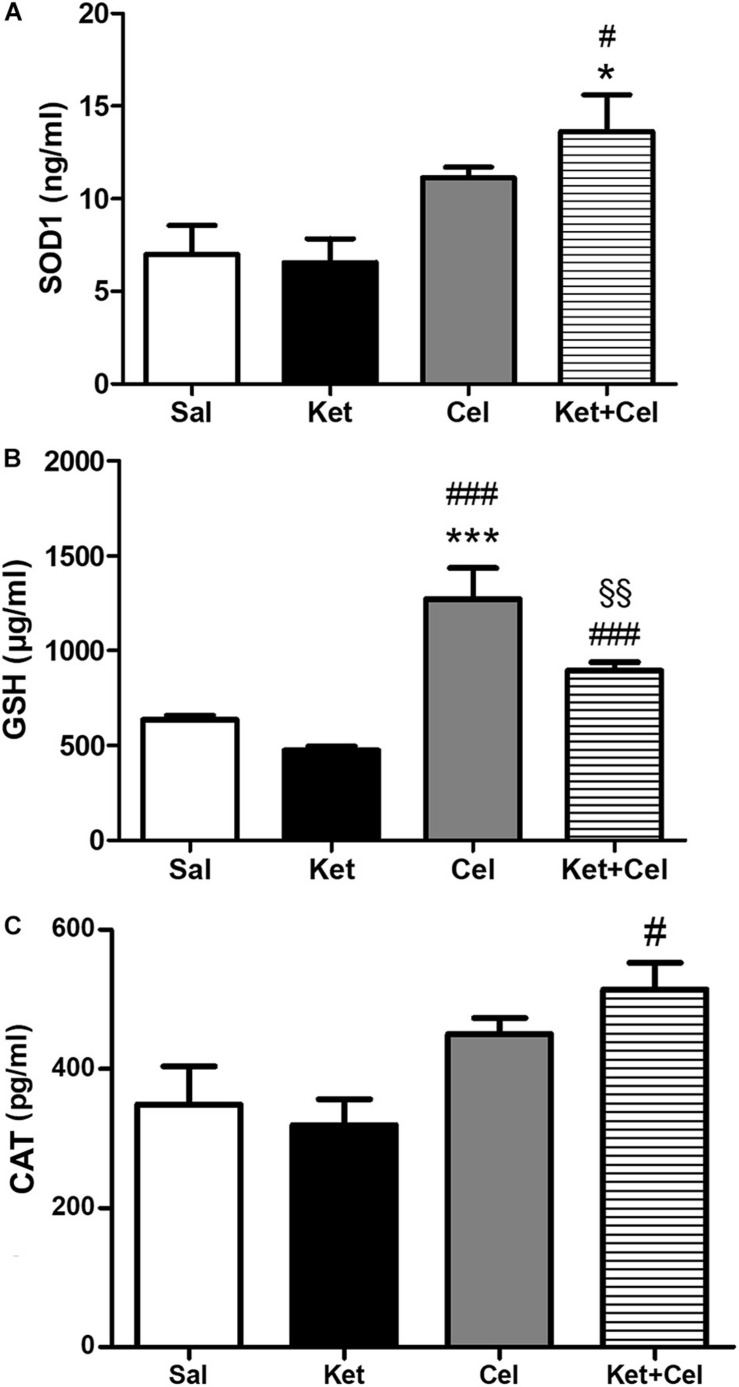
Effects of early celastrol administration on SOD1, GSH, and CAT levels in the PFC of adult mice, administered with ketamine in postnatal life. **(A)** SOD1 levels (ng/ml) in the PFC of 10 weeks mice receiving saline (Sal, *n* = 5) or ketamine (Ket, *n* = 5) or celastrol (Cel, *n* = 4) or ketamine + celastrol (Ket + Cel, *n* = 4) at PNDs 7, 9, and 11. One way ANOVA, followed by Tukey’s Multiple Comparison *post hoc* test, *F*_(3,14)_ = 5.318, *p < 0.05 Ket + Cel vs Sal, ^#^*p* < 0.05 Ket + Cel vs Ket. **(B)** GSH levels (μg/ml) in the PFC of 10 weeks mice receiving saline (Sal, *n* = 3) or ketamine (Ket, *n* = 7) or celastrol (Cel, *n* = 3) or ketamine + celastrol (Ket + Cel, *n* = 7) at PNDs 7, 9, and 11. One way ANOVA, followed by Tukey’s Multiple Comparison *post hoc* test, *F*_(3,16)_ = 29.89, ***p < 0.001 Cel vs Sal, ^###^*p* < 0.001 Cel vs Ket and Ket + Cel vs Ket, ^§§^ *p* < 0.01 Ket + Cel vs Cel. **(C)** CAT levels (pg/ml) in the PFC of 10 weeks mice receiving saline (Sal, *n* = 4) or ketamine (Ket, *n* = 5) or celastrol (Cel, *n* = 3) or ketamine + celastrol (Ket + Cel, *n* = 5) at PNDs 7, 9, and 11. One way ANOVA, followed by Tukey’s Multiple Comparison *post hoc* test, *F*_(3,13)_ = 5.095, ^#^*p* < 0.05 Ket + Cel vs Ket.

**FIGURE 4:**
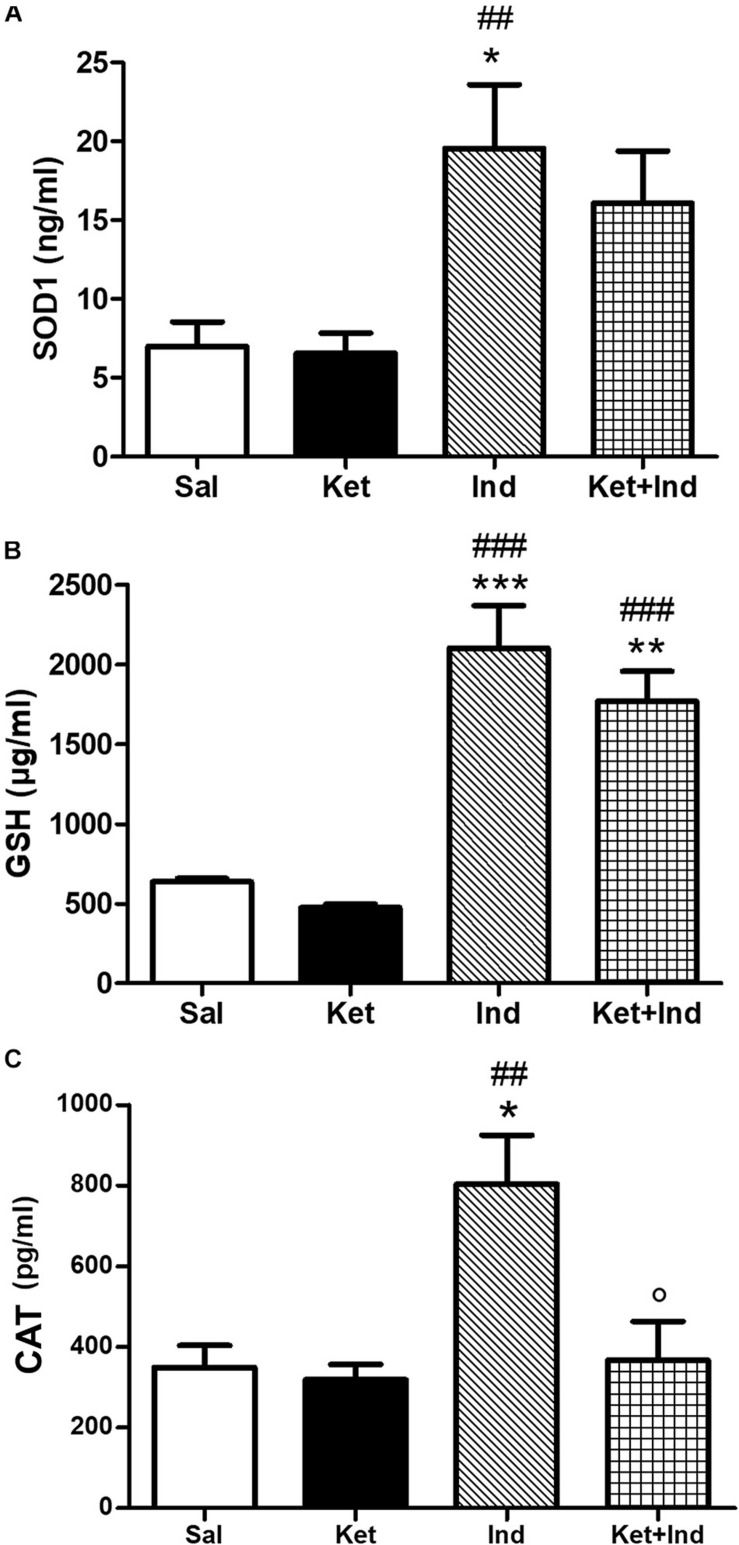
Effects of early indomethacin administration on SOD1, GSH, and CAT levels in the PFC of adult mice, administered with ketamine in postnatal life. **(A)** SOD1 levels (ng/ml) in the PFC of 10 weeks mice receiving saline (Sal, *n* = 5) or ketamine (Ket, *n* = 5) or indomethacin (Ind, *n* = 3) or ketamine + indomethacin (Ket + Ind, *n* = 3). One way ANOVA, followed by Tukey’s Multiple Comparison *post hoc* test, *F*_(3,12)_ = 7.715, *p < 0.05 Ind vs Sal, ^##^*p* < 0.01 Ind vs Ket. **(B)** GSH levels (μg/ml) in the PFC of 10 weeks mice receiving saline (Sal, *n* = 3) or ketamine (Ket, *n* = 7) or indomethacin (Ind, *n* = 5) or ketamine + indomethacin (Ket + Ind, *n* = 5). One way ANOVA, followed by Tukey’s Multiple Comparison *post hoc* test, *F*_(3,16)_ = 25.59, ***p < 0.001 Ind vs Sal, **p < 0.01 Ket + Ind vs Sal, ^###^*p* < 0.001 Ind and Ket + Ind vs Ket. **(C)** CAT levels (pg/ml) in the PFC of 10 weeks mice receiving saline (Sal, *n* = 4) or ketamine (Ket, *n* = 5) or indomethacin (Ind, *n* = 5) or ketamine + indomethacin (Ket + Ind, *n* = 5). One way ANOVA, followed by Tukey’s Multiple Comparison *post hoc* test, *F*_(3,15)_ = 7.200, *p < 0.05 Ind vs Sal,^##^*p* < 0.01 Ind vs Ket, *p* < 0.05 Ind + Ket vs Ind.

To investigate the possible impact of early celastrol or indomethacin administration on antioxidant enzyme expression following ketamine exposure in postnatal life, we quantified levels of SOD1, CAT, and GSH in the PFC of adult mice. Whereas comparable SOD1 amount was detected among saline, ketamine and celastrol-treated animals, significant increased expression of this antioxidant enzyme was observed in ketamine + celastrol-treated animals with respect to both saline and ketamine groups (Figure 3A, One way ANOVA, followed by Tukey’s Multiple Comparison *post hoc* test, *F*_(3,14)_ = 5.318, *p* < 0.05). Early indomethacin treatment *per se* resulted in an increased SOD1 expression with respect to both saline and ketamine-administered mice (Figure 4A, One way ANOVA, followed by Tukey’s Multiple Comparison *post hoc* test, *F*_(3,12)_ = 7.715, *p* < 0.05 and *p* < 0.01).

No significant differences in GSH levels were detected between early ketamine and saline-treated animals. Increased amount of this enzyme was observed in celastrol-treated animals with respect to the saline and ketamine groups and in animals exposed to ketamine + celastrol compared to ketamine and celastrol-treated mice (Figure 3B, One way ANOVA, followed by Tukey’s Multiple Comparison *post hoc* test, *F*_(3,16)_ = 29.89, *p* < 0.01, and *p* < 0.001). Administration of indomethacin in early postnatal life, alone or in combination with ketamine, was able to elevate GSH expression with respect to controls and ketamine-treated mice (Figure 4B, One way ANOVA, followed by Tukey’s Multiple Comparison *post hoc* test, *F*_(3,16)_ = 25.59, *p* < 0.01, *p* < 0.001).

Whereas early ketamine exposure did not affect CAT levels in the PFC of adult mice, increased levels of this antioxidant enzyme were detected in ketamine + celastrol-treated animals compared to ketamine groups (Figure 3C, One way ANOVA, followed by Tukey’s Multiple Comparison *post hoc* test, *F*_(3,13)_ = 5.095, *p* < 0.05). Levels of this enzyme were enhanced following early administration of indomethacin compared to saline or ketamine-treated mice and this was prevented by ketamine + indomethacin injection (Figure 4C, One way ANOVA, followed by Tukey’s Multiple Comparison *post hoc* test, *F*_(3,15)_ = 7.200, *p* < 0.05 and *p* < 0.01).

### Effects of Early Celastrol or Indomethacin Administration on Pro-inflammatory Cytokines in the PFC of Adult Mice Treated With Ketamine in Postnatal Life

**FIGURE 5:**
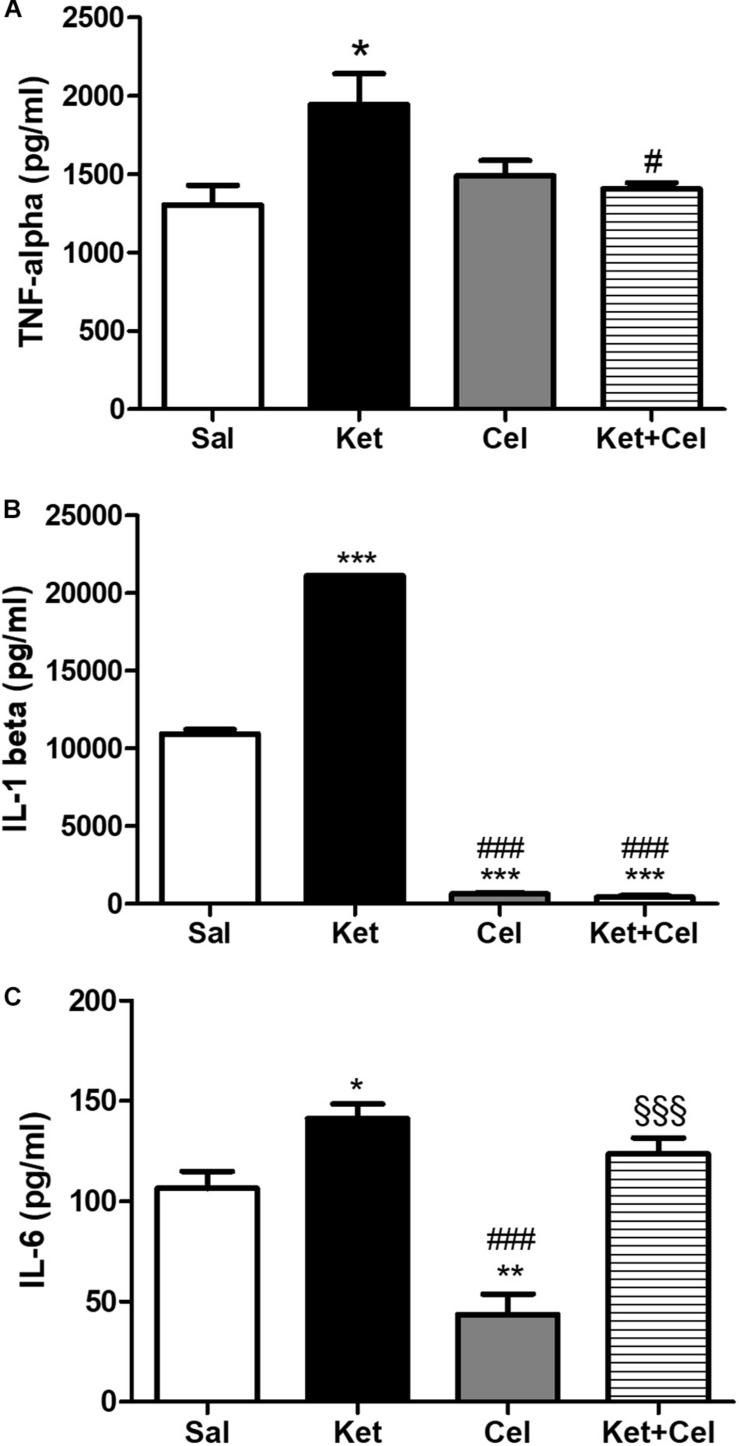
Effects of early celastrol administration on TNF-ɑ and IL-1ß levels in the PFC of adult mice, administered with ketamine in postnatal life. **(A)** TNF-ɑ levels (pg/ml) in the PFC of 10 weeks mice receiving saline (Sal, *n* = 4) or ketamine (Ket, *n* = 5) or celastrol (Cel, *n* = 4) or ketamine + celastrol (Ket + Cel, *n* = 5) at PNDs 7, 9, and 11. One way ANOVA, followed by Tukey’s Multiple Comparison *post hoc* test, *F*_(3,14)_ = 4.708; *p < 0.05 Ket vs Sal; ^#^*p* < 0.05 Ket + Cel vs Ket. **(B)** IL-1ß levels (pg/ml) in the PFC of 10 weeks mice receiving saline (Sal, *n* = 3) or ketamine (Ket, *n* = 4) or celastrol (Cel, *n* = 3) or ketamine + celastrol (Ket + Cel, *n* = 4) at PNDs 7, 9, and 11. One way ANOVA, followed by Tukey’s Multiple Comparison *post hoc* test, *F*_(3,10)_ = 5538, ***p < 0.001 Ket, Cel and Ket + Cel vs Sal; ^###^*p* < 0.001 Cel and Ket + Cel vs Ket. **(C)** IL-6 levels (pg/ml) in the PFC of 10 weeks mice receiving saline (Sal, *n* = 5) or ketamine (Ket, *n* = 5) or celastrol (Cel, *n* = 3) or ketamine + celastrol (Ket + Cel, *n* = 5) at PNDs 7, 9, and 11. One way ANOVA, followed by Tukey’s Multiple Comparison *post hoc* test, *F*_(3,14)_ = 20.08, *p < 0.05 Ket vs Sal; **p < 0.01 Cel vs Sal, ^###^*p* < 0.001 Cel vs Ket, ^§§§^ *p* < 0.001 Ket + Cel vs Cel.

**FIGURE 6:**
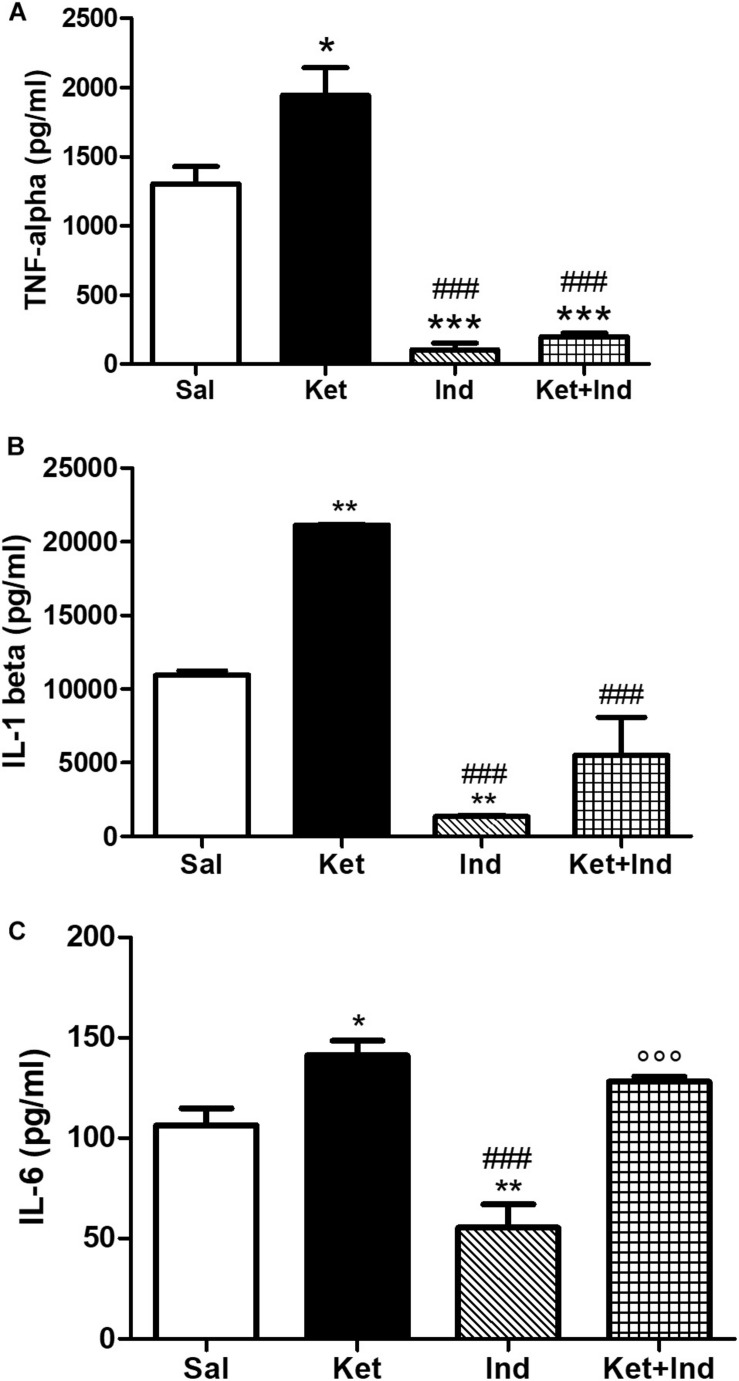
Effects of early indomethacin administration on TNF-ɑ and IL-1ß levels in the PFC of adult mice, administered with ketamine in postnatal life. **(A)** TNF-ɑ levels (pg/ml) in the PFC of 10 weeks mice receiving saline (Sal, *n* = 4) or ketamine (Ket, *n* = 5) or indomethacin (Ind, *n* = 4) or ketamine + indomethacin (Ket + Ind, *n* = 4) at PNDs 7, 9, and 11. One way ANOVA, followed by Tukey’s Multiple Comparison *post hoc* test, *F*_(3,13)_ = 45.20, *p < 0.05 Ket vs Sal, ***p < 0.001 Ind and Ket + Ind vs Sal, ^###^*p* < 0.001 Ind and Ket + Ind vs Ket. **(B)** IL-1ß levels (pg/ml) in the PFC of 10 weeks mice receiving saline (Sal, *n* = 3) or ketamine (Ket, *n* = 4) or indomethacin (Ind, *n* = 4) or ketamine + indomethacin (Ket + Ind, *n* = 4) at PNDs 7, 9, and 11. One way ANOVA, followed by Tukey’s Multiple Comparison *post hoc* test, *F*_(3,11)_ = 39.92, **p < 0.01 Ket and Ind vs Sal, ^###^*p* < 0.001 Ind and Ket + Ind vs Ket. **(C)** IL-6 levels (pg/ml) in the PFC of 10 weeks mice receiving saline (Sal, *n* = 5) or ketamine (Ket, *n* = 5) or indomethacin (Ind, *n* = 4) or ketamine + indomethacin (Ket + Ind, *n* = 3) at PNDs 7, 9, and 11. One way ANOVA, followed by Tukey’s Multiple Comparison *post hoc* test, *F*_(3,13)_ = 18.41, *p < 0.05 Ket vs Sal, **p < 0.01 Ind vs Sal, ^###^*p* < 0.001 Ind vs Ket, °°*p* < 0.001 Ket + Ind vs Ind.

To evaluate possible effects of early celastrol or indomethacin administration on pro-inflammatory cytokines following ketamine exposure in postnatal life, we quantified levels of TNF-ɑ, IL-1ß, and IL-6 in the PFC of adult mice. Whereas no significant differences in TNF-ɑ amount was detected among saline and celastrol treatments, ketamine administration at PNDs 7, 9, and 11 resulted in TNF-ɑ elevations, which were prevented by early celastrol administration (Figure 5A, One way ANOVA, followed by Tukey’s Multiple Comparison *post hoc* test, *F*_(3,14)_ = 4.708, *p* < 0.05). Indomethacin, both *per se* and concomitantly administered with ketamine, was able to decrease TNF-ɑ levels compared to controls and ketamine-exposed mice (Figure 6A, One way ANOVA, followed by Tukey’s Multiple Comparison *post hoc* test, *F*_(3,13)_ = 45.20, *p* < 0.05, and *p* < 0.001).

Cortical levels of IL-1ß in adult mice were enhanced following ketamine administration in early life compared to saline-treated animals. Celastrol treatment was able to significantly decrease amount of these pro-inflammatory cytokines in the PFC and this was also observed when it was administered with ketamine (Figure 5B, One way ANOVA, followed by Tukey’s Multiple Comparison *post hoc* test, *F*_(3,10)_ = 5538, *p* < 0.001). Despite IL-1ß amount was significantly lowered by indomethacin *per se* compared to both saline and ketamine-treated mice, levels of this cytokine following early ketamine + indomethacin administration were comparable to the ones detected in the saline group, although still significantly decreased with respect to ketamine-exposed mice (Figure 6B, One way ANOVA, followed by Tukey’s Multiple Comparison *post hoc* test, *F*_(3,11)_ = 39.92, *p* < 0.01, and *p* < 0.001).

Early ketamine treatment resulted in increased IL-6 levels in the PFC of adult mice. Pups that received only celastrol or indomethacin showed, at adulthood, a significant decrease of this cytokine compared to both saline and ketamine-administered animals. However, in mice concomitantly injected with ketamine, no significant differences were detected compared to animals that received saline or ketamine in early life (Figure 5C, One way ANOVA, followed by Tukey’s Multiple Comparison *post hoc* test, *F*_(3,14)_ = 20.08, *p* < 0.05, *p* < 0.01, and *p* < 0.001 and Figure 6C, One way ANOVA, followed by Tukey’s Multiple Comparison *post hoc* test, *F*_(3,13)_ = 18.41, *p* < 0.05, *p* < 0.01, and *p* < 0.001).

## Discussion

In this work, we demonstrated that administration of subanesthetic doses of ketamine at PNDs 7, 9, and 11 caused increased ROS production in the PFC of adult mice. Supporting these findings, previous observations, obtained on the same animal model, showed both early and persistent increased levels of 8-hydroxydeoxyguanosine (8OHdG), an indirect marker of oxidative stress, and NOX2, a ROS-producing enzyme, in the same brain region (Schiavone et al., 2020). Accordingly, it has been reported that, although oxidative stress contributes to the physiological postnatal brain development in rodents, the effects of increased ROS levels on the CNS, following an external insult, might be revealed later in life (Wilhelm et al., 2016). Moreover, antioxidant treatment with N-acetyl cysteine in mice could prevent cognitive and behavioral dysfunctions at adulthood, resulting from ketamine administration at PNDs 7, 9, and 11 (Phensy et al., 2017).

Here, we also observed enhanced lipid peroxidation after early ketamine exposure. In good agreement with this finding, ketamine-induced increase in MDA content in the cortex of young rodents, associated with elevations in levels of indirect markers of oxidative stress were previously reported (Cheung and Yew, 2019). Interestingly, changes in brain lipid peroxidation have been described during early postnatal development, with a physiological decrease in adult animals (Galkina et al., 2009). Indeed, during the neonatal period, brain has been reported to have low peroxidation potential corresponding to the rapid phase of cell proliferation (Pushpendran et al., 1994). Dysfunctions of this process, induced by an external trigger, such as the exposure to NMDA-R antagonists, have been shown to result in the persistence of high levels of lipid peroxidation at adulthood, contributing to the development of psychotic-like neuropathological and behavioral dysfunctions in rodents (de Carvalho Cartágenes et al., 2019), as well as neuropsychiatric disorders in humans (Joshi and Pratico, 2014; Romano et al., 2017).

An important finding of our study consists in the lack of significant differences in cortical amounts of antioxidant enzymes between early ketamine-treated mice and controls. This result might appear contrasting with preclinical evidence describing, instead, a decreased activity of SOD and CAT in the PFC of ketamine-treated rats (de Oliveira et al., 2009), as well as a reduction of GSH concentration (Abdel-Salam et al., 2015). However, in these previously published studies, ketamine exposure occurred in adult life. Furthermore, the lack of differences in levels of antioxidant enzymes observed in our experimental conditions might be also interpreted as a long-term dysfunction, induced by early ketamine exposure, of the physiological roles of the antioxidant system in controlling ROS damage and in regulating ROS signaling (Wang et al., 2018).

Here, we also demonstrated that early celastrol treatment prevented ketamine-induced increased lipid peroxidation and ROS production in the PFC of adult mice. Different mechanisms of action have been proposed to describe celastrol pharmacological effects. Among these, the induction of the expression of neuroprotective factors, the block of ROS-induced cellular apoptosis and the decrease of oxidative stress have been reported (Chen et al., 2018). In particular, the reduction of oxidative stress also occurs via the inhibition of the NADPH oxidase NOX enzymes with an increased potency against NOX1 and NOX2 isoforms, resulting in the lack of the functional association between the cytosolic subunits and the membrane flavocytochrome of these enzymes (Jaquet et al., 2011; Tarafdar and Pula, 2018). The effect of celastrol on ketamine-induced increase in MDA and ROS levels observed in the present study is in line with previous evidence obtained on the same animal model, where celastrol administration in postnatal life was found to prevent ketamine-induced elevations in cerebellar expression of 8OHdG, a marker of oxidative damage to DNA, and of the ROS producing enzyme NADPH oxidase NOX1 (Schiavone et al., 2019). Intriguingly, in our experimental conditions, the reduction of cortical ROS amount and lipid peroxidation induced by early celastrol administration was accompanied by increased levels of SOD1, GSH and CAT with respect to mice receiving only ketamine in postnatal life. Supporting our hypothesis, previous preclinical evidence have reported that celastrol could attenuate oxidative damage by increasing levels and activity of SOD, GSH, glutathione peroxidase, glutathione reductase, and CAT (Shaker et al., 2014; Wang et al., 2014; Boran et al., 2019; Gao et al., 2020). Although still speculative, the effects of celastrol on cortical ROS levels and lipid peroxidation, at least in our experimental conditions, might be explained by a concomitant action of this compound on ROS production and their degradation, finally resulting in the recovery of the redox balance, early altered by ketamine. Indeed, it might be hypothesized that celastrol administration may inhibit ketamine-induced increase in NOX2 expression observed in the PFC of mice pups and, consequently, the persistent elevations of this enzyme in adult life (Schiavone et al., 2020). On the other side of the redox balance, celastrol exposure in the early phases of postnatal life might result in the recovery of the physiological role of the antioxidant system in response to ketamine-induced oxidative stress elevations. Hence, ketamine-induced increase of ROS production and consequent lipid peroxidation might be prevented by the synergistic action of these two hypothesized mechanisms.

We also showed that early administration of indomethacin *per se* could decrease MDA levels in the PFC of adult mice treated with ketamine in early life. This finding might appear in contrast with a previous report showing an aggravation of lipid peroxidation, in terms of increased MDA levels, in newborn rats with hypoxic-ischemic cerebral injury following indomethacin administration (Taskin et al., 2009). However, the result obtained in our experimental conditions might be explained by both the different time point at which this parameter (adult life) was evaluated and the kind of insult impacting on the developing brain. Indeed, in this regard, previous evidence reported a beneficial effect of indomethacin in reducing peripheral and central MDA levels following rat exposure to other neurotoxic insult, such as CCl_4_ (Kadiiska et al., 2005). Moreover, cortical increase of lipid peroxidation, induced in rats by an infectious insult, was also found to be reduced following intraperitoneal indomethacin administration (Guzman et al., 2018). However, we cannot totally exclude that indomethacin *per se* could induce an increase in MDA levels in the early phases of postnatal life, thus stimulating in the developing brain the activation of neuroprotective mechanisms that may result in the decreased lipid peroxidation we observed in adult mice.

Interestingly, indomethacin *per se* induced elevations in levels of all the considered antioxidant enzymes compared to both controls and early ketamine-exposed mice. Thus, it might be hypothesized that increased antioxidant defense might be a possible mechanism underlying indomethacin effects on cortical lipid peroxidation. Supporting our hypothesis, COX inhibition has been reported to significantly improve antioxidant defense both at peripheral and central levels (Kumar et al., 2011; Ahmed et al., 2014). Concomitant administration of ketamine and indomethacin could prevent the cortical increase in ROS amount and in MDA levels induced by early ketamine exposure, suggesting a neuroprotective role of this compound against the impact that a neurodetrimental insult might have on the developing brain. In support of this hypothesis, indomethacin has been reported to prevent the loss of neurogenesis markers following a neurotoxic insult, i.e., ethanol in adolescent rodents (Vetreno et al., 2018). Furthermore, indomethacin was able to regulate the peripheral expression of neurotrophins, such as BDNF and NGF (Kemi et al., 2006; Hochstrasser et al., 2013). Hence, it can be hypothesized that this might also happen at central level following an early neurotoxic insult affecting CNS development. Moreover, clinical evidence reported the use of indomethacin as neuroprotective strategy to prevent the later consequences of neonatal brain injury (Favrais et al., 2014), via the strengthening of the immature blood-brain barrier (Sims, 2012).

In this manuscript, it is also showed that ketamine administration in early life stages caused an enhancement of proinflammatory cytokines, i.e., TNF-α, IL-1ß, and IL-6, at adulthood. In good agreement with our findings, previous preclinical and clinical reports highlighted a crucial role of early and persistent cortical neuroinflammation in the development of psychotic-like symptoms in rodents (Schiavone et al., 2017; Ben-Azu et al., 2019; Kogan et al., 2019) as well as of schizophrenia in humans (Zhang et al., 2016; Barron et al., 2017). Importantly, it has been reported that dysregulation of the redox, immune and glutamatergic systems, induced by NMDA-R antagonists, including ketamine, especially when it occurs during brain development, represents a “central hub” in schizophrenia pathophysiology (Steullet et al., 2016). In line with this concept, in the same animal model, together with increased levels of pro-inflammatory cytokines, we do observe increased cortical ROS amount and lipid peroxidation and we previously demonstrated early and persistent increase of oxidative damage to DNA as well as alterations of NADPH expression in the same brain region (Schiavone et al., 2020).

In our experimental conditions, together with an effect of celastrol *per se* on IL-1ß and of indomethacin *per se* on TNF-α and IL-1ß, we also found that early celastrol or indomethacin administrations were able to prevent ketamine-induced elevations in TNF-α and IL-1ß in PFC of adult mice, suggesting a possible protective role of these two compounds against the possible long-lasting detrimental effects exerted by early neuroinflammation on the developing brain (Franceschini and Zusso, 2019). This result should also be considered in the light of previous evidence showing a reduction of microglia activation following celastrol (Dai et al., 2019) or indomethacin (Lopes et al., 2016) administration, as well as a strict interrelation between TNF-α and IL-1ß and redox dysregulation in CNS disorders (Fischer and Maier, 2015; Schiavone and Trabace, 2017). Indeed, it might be hypothesized that ketamine administration in postnatal life may cause microglia activation, with consequent release of TNF-α and IL-1ß which, in turn, induce ROS production, further sustaining neuroinflammation and neuronal damage. In addition, the possible inhibition of ketamine-induced enhancement of microglial NADPH oxidase NOX2 by celastrol administration might also play a key role in this process. Hence, in this regard, it has been highlighted that NOX2 activation in microglia exerts neurotoxic effects via extracellular ROS production as well as the initiation of microglia redox signaling, finally resulting in the amplification of the pro-inflammatory response (Surace and Block, 2012).

Despite the decrease in cortical IL-6 levels detected following celastrol or indomethacin treatments alone compared to both controls and ketamine-exposed mice, these two compounds, administered concomitantly with ketamine, could not prevent elevations of this pro-inflammatory cytokine in postnatal life. This result should be considered in the light of the physiological expression of IL-6 and its receptor in rodent cortex during postnatal development (Gadient and Otten, 1994), as well as of the central role reported for this cytokine in the promotion of postnatal murine CNS development, most likely being perturbations in its levels the cause of long-lasting and irreversible damage (Storer et al., 2018). However, we cannot totally exclude that a possible effects of celastrol or indomethacin on ketamine-induced dysfunctions of IL-6 levels might have been detected at different time points from ketamine exposure, such as during mice adolescence or later than 10 weeks of life. Further investigations are certainly needed in this sense, also considering the physiological link existing between IL-6 and anti-inflammatory cytokines (Ropelle et al., 2010), as well as the role of the pro-inflammatory/anti-inflammatory balance in neurodevelopmental-related mental disturbances (Ratnayake et al., 2013).

With respect to a possible translation of the results of the present study to clinics, a limitation consists in the fact that, in animals, pharmacological treatments were initiated at the same time of ketamine administration. Indeed, this same therapeutic strategy cannot be directly translated into the clinical setting, because of the impossibility to identify in humans the exact time of the neurotoxic insult.

In conclusion, our data suggest that both the enhancement of antioxidant defense, reducing cerebral oxidative stress and inhibition of inflammatory pathways, may represent a suitable therapeutic approach preventing psychotic-like disturbances resulting from the impact of neurotoxic insult during crucial phases of brain maturation.

## Data Availability Statement

The raw data supporting the conclusions of this article will be made available by the authors, without undue reservation.

## Ethics Statement

The animal study was reviewed and approved by the Italian Ministry of Health (approval number 679/2017-PR, protocol n. B2EF8.17).

## Author Contributions

MB, PT, LT, SS, and MGM designed the research. MB, PT, SD, SS, and MGM performed the research. MB, PT, SS, and MGM analyzed the data. MB, PT, LT, and SS wrote the manuscript. MB, PT, SD, LT, SS, and MGM revised the manuscript. All authors contributed to the article and approved the submitted version.

## Conflict of Interest

The authors declare that the research was conducted in the absence of any commercial or financial relationships that could be construed as a potential conflict of interest.
